# Characterization of Small Molecules Inhibiting the Pro-Angiogenic Activity of the Zinc Finger Transcription Factor Vezf1

**DOI:** 10.3390/molecules23071615

**Published:** 2018-07-03

**Authors:** Ming He, Qianyi Yang, Allison B. Norvil, David Sherris, Humaira Gowher

**Affiliations:** 1 Department of Biochemistry, Purdue University, West Lafayette, IN 47907, USA; he261@purdue.edu (M.H.); qianyiyang@wustl.edu (Q.Y.); anorvil@purdue.edu (A.N.); 2 Department of Anesthesiology, Washington University School of Medicine, 660 S Euclid Ave, St. Louis, MO 63110, USA; 3 GenAdam Therapeutics, Inc., 37 Neillian Crescent, Jamaica Plain, MA 02130, USA; dsherris@genadamthera.com; 4 Purdue University Center for Cancer Research, Purdue University, West Lafayette, IN 47907, USA

**Keywords:** Vezf1, angiogenesis, vascular biology, endothelial cells, MSS31, tube formation, small molecule inhibitors computational modeling

## Abstract

Discovery of inhibitors for endothelial-related transcription factors can contribute to the development of anti-angiogenic therapies that treat various diseases, including cancer. The role of transcription factor Vezf1 in vascular development and regulation of angiogenesis has been defined by several earlier studies. Through construction of a computational model for Vezf1, work here has identified a novel small molecule drug capable of inhibiting Vezf1 from binding to its cognate DNA binding site. Using structure-based design and virtual screening of the NCI Diversity Compound Library, 12 shortlisted compounds were tested for their ability to interfere with the binding of Vezf1 to DNA using electrophoretic gel mobility shift assays. We identified one compound, T4, which has an IC50 of 20 μM. Using murine endothelial cells, MSS31, we tested the effect of T4 on endothelial cell viability and angiogenesis by using tube formation assay. Our data show that addition of T4 in cell culture medium does not affect cell viability at concentrations lower or equal to its IC 50 but strongly inhibits the network formation by MSS31 in the tube formation assays. Given its potential efficacy, this inhibitor has significant therapeutic potential in several human diseases.

## 1. Introduction

Vascular Endothelial Zinc Finger 1 (Vezf1), previously named Bgp1, was discovered as a protein binding to a poly (dG) sequence present in the neighborhood of chicken β-globin promoter [1,2]. Vezf1 belongs to the family of Kruppel-like zinc finger protein that contains six C2H2 class zinc finger motifs (~99% identical between homologues. It is about 65 kd protein and recognizes long strings of poly (dG).poly (dC) (‘G-strings’). Biochemical studies revealed that the minimal binding site of Vezf1 is a (dG)^7^ string, but it also binds to a bipartite poly G string containing a (dG)^6^ and (dG)^4^ separated by three to four nucleotides present in the upstream hypersensitive site 5′HS4 of chicken β-globin locus [3]. Interestingly, poly G tracts are highly prevalent in CpG islands in mammalian genomes resulting in a number of putative binding sites in these regions. Similar to some other zinc finger proteins such as CTCF, Vezf1 is conserved on among vertebrates and the proteins are near identical between mouse and human [4,5].

The expression of Vezf1 is highly restricted in vascular endothelium during embryogenesis. Targeted inactivation of Vezf1 gene in mice causes embryonic lethality and it acts in a dosage dependent fashion to regulate the development of blood and lymphatic vascular system [6,7]. Embryonic stem cells (ESCs) derived from Vezf1^−/−^ embryos differentiated into embryoid bodies were shown to have defect sprouting angiogenesis. However, the loss of Vezf1 in embryos had no significant effect on the expression of pioneer factors that regulate vasculogenesis including VEGF-A-A (Vascular Endothelial Growth Factor) and ET-1, indicating a complex mechanism [8]. Our genome-wide gene expression analysis of the Vezf1^−/−^ ESCs showed a significant high expression of the antiangiogenic factor Cited2/Mrg1 [9]. Using an in vitro endothelial cell (EC) differentiation and tube formation as model system for angiogenesis, our data showed that some of the differentiation and angiogenesis defects in Vezf1^−/−^ ECs could be rescued by reducing the Cited2 expression in these cells [10]. Other studies have shown the function of Vezf1 in regulation of adult angiogenesis by activating pro-angiogeneic genes including microtubule turnover protein, stathmin/OP18, and metallothionein 1 (MT1) [7,11,12]. Haploinsufficient Vezf1 mice exhibit reduced angiogenic response to injury. Using primary human endothelial cells (BVECs), Vezf1 was shown to be functionally linked with Rho B which is a small GTPase known to be involved in angiogenesis during post-natal retinal development [13,14]. To understand the mechanism of Vezf1, our previous studies showed a major loss of DNA methylation genome-wide with a concomitant reduction in the expression of a major DNA methyltransferase Dnmt3b in Vezf1^−/−^ ESCs [15]. We further showed that Vezf1 binding sites overlap with the RNA polymerase 2 pausing sites genome-wide and interacts with chromatin modifying proteins [9]. However the details of Vezf1 mechanism and how it impacts gene expression in early development or in somatic cells in not known.

Formation of vascular system is the most critical and earliest step in development and is a critical requirement for tumor progression and metastasis [16,17]. Several studies have led to the identification of many transcription factors that regulate angiogenesis but most of them are not endothelial specific thus making it difficult to use them as targets for anti-cancer drug development [18,19]. Vezf1 is unique among endothelial factors since during development its function is restricted to vascular system thus making it an attractive target for cancer therapeutics [6]. Although Vezf1 null mutants are embryonic lethal, Vezf1 null embryonic stem cell derived teratocarcinomas were able to differentiate into each of the three germ layer. However, these tumors showed decreased proliferative ability and delayed differentiation [8]. Currently approved angiogenesis inhibitors target the VEGF pathway by direct inhibition of VEGF-A (bevacizumab) or VEGF-A receptors (sunitinib and sorafenib), and few explore alternatives to this direct inhibition [20]. New endothelial-related transcription factors can contribute to the development of antiangiogenic therapies that treat diseases ranging from cardiovascular disease to cancer. The need for new cancer therapeutics and the necessity to understand the complex mechanisms involved in regulation of cell-type specific genes has increased the demand for comprehensive characterization of small molecule inhibitors against factors such as Vezf1 that can potentially work in a restricted fashion and block angiogenesis and hyper-vascularization in diseased state.

In the present study we used recombinant Vezf1 protein to characterize 12 compounds that could potentially inhibit its binding to DNA. We found T4 to block Vezf1-DNA binding at low μM concentration. We tested the cellular toxicity and activity of T4 by using MSS31 endothelial cells. We show a specific inhibition of tube formation by MSS31 cells which are viable when treated with a dose equal to IC50 of T4. We therefore report an effective compound that can potentially block angiogenesis without affecting cell viability.

## 2. Results

### 2.1. Computational Modelling of Vezf1 Structure, Evaluation of Potential Binding Sites and Design of Small Molecule Inhibitors

**Figure 1:**
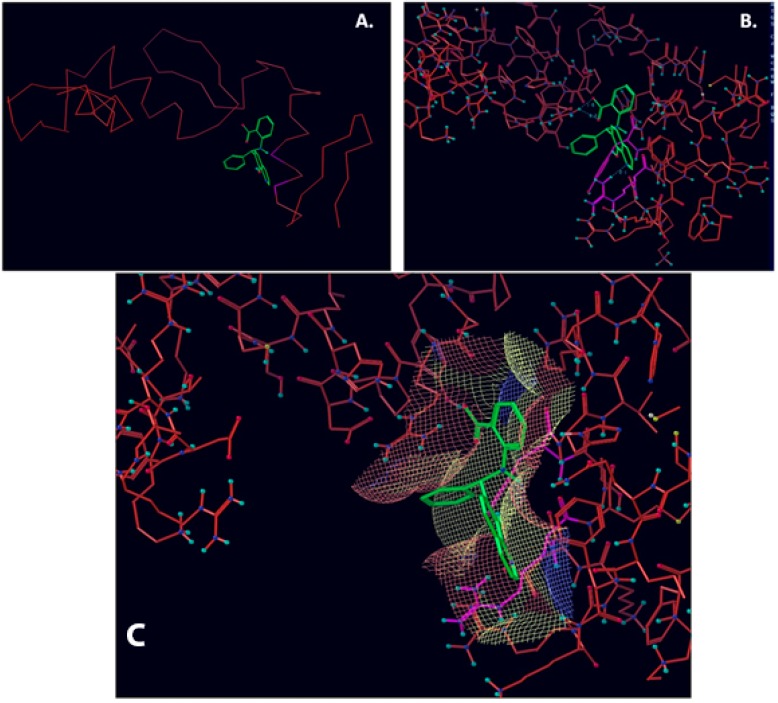
NSC1012 docked into Vezf1 Model binding site. (**A**) The alpha carbon trace and (**B**) complete model. (**C**) NSC1012 docked into Model binding site with the solvent accessible surface: yellow covers hydrophobic areas of the binding site, red, hydrogen bond acceptor, and blue hydrogen bond donor.

**Figure 2:**
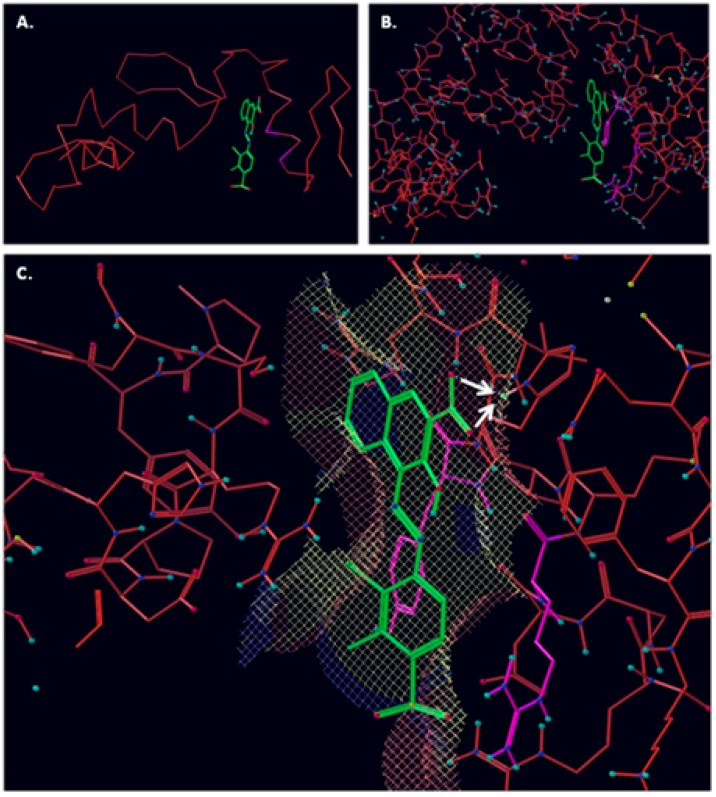
NSC16087 docked into Model II binding site. In (**A**) alpha carbon trace, in (**B**) complete model and in (**C**) NSC16087 docked into Model binding site with the accessible surface mesh. Hydrogen bonds to His 48 are shown with white arrows.

Computational modeling and design of small molecules was done at Vasculomedics Inc. (Boston, MA, USA). A model of the zinc finger, Vezf1 that would compare favorably to the experimentally determined structures of other zinc fingers was determined. Using high resolution DNA bound structure of Zinc Finger 268 and 1AAY [21], the structure was further refined using Monte Carlo searching (see Figure S1). VasculoMedics, Inc. had previously tested a few lead compounds that inhibit Vezf1 DNA binding using previously designed model of Vezf1. Docking tools of FLO (McDock+) were used to dock these known ligands (see Figure 1 and Figure 2). New compounds were designed such that they incorporate only minimal changes from the lead compounds thus using docking experiments of lead compounds as a guide. The de novo program AlleGrow was used to discover novel side chains to replace those of the leads. Each designed compound was docked and evaluated in the model. Those structures were selected which formed the most favorable interactions and had good energy profiles. After evaluation of several lead compounds for complexity in synthesis, six compounds were selected and synthesized at MedChem Partners. Next, a major virtual screening program was performed in order to find compounds at the NCI Diversity Compound Library.

### 2.2. Virtual Screening

We used major virtual screening program to find compounds that could be obtained from the NCI Diversity Compound Library. The first step for virtual screening was to identify the docking protocol, which gave the best result when docking compounds of known activity. Therefore, two docking algorithms were tested by docking all compounds. The methods tested included:

(1) Sdock+ is a very fast docking algorithm that uses a novel method for generating vast numbers of conformers in the target-binding site. The best scoring conformers are energy minimized in the binding site.
(2) McDock+ uses a Monte Carlo algorithm for generating new conformers. Conformers generated by McDock+ are optimized with energy minimization and the FLO scoring function. The calculations are much more time consuming than Sdock+, however, often the Mcdock+ results are more reliable.
(3) SDock+ followed by McDock+ Conformers found by SDock+ served as a starting point for McDock+.

**Figure 3:**
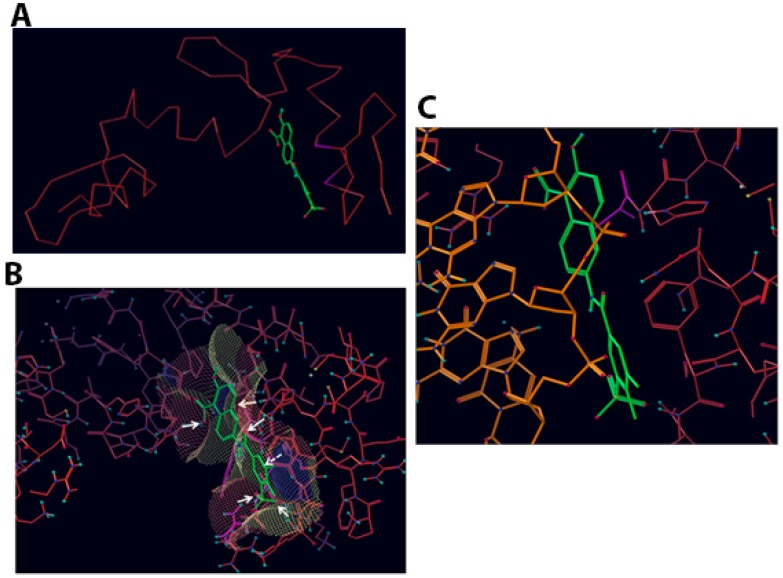
Compound T4 docked into Vezf1 Model binding site. (**A**) Only the alpha carbon trace is shown to indicate how T4 fits between two fingers. (**B**) Compound T4 docked into binding site with the accessible surface mesh. Arrows show important interactions with solid arrows indicating hydrogen bonds and dotted lines show stacking interactions between the compounds phenyl and phenyl ring of PHE123. (**C**) Compound T4 (green carbon atoms) docked into binding site (red carbon atoms). The structure 1AAY of the zinc finger 268 with DNA has been superimposed. Only DNA (orange carbon atoms) is shown.

Each method was evaluated by determining the rank order obtained for the three lead compounds as shown in Table 1. SDock+ alone was much faster with results as good as Sdock+ followed by McDock+ and better than straight McDock+. Hence, SDock+ was used to screen the NCI Diversity Compound Library. Upon finding positive compounds selected from virtual screening in the NCI database, each compound was evaluated in the model (see Figure 3). 

Those forming the most favorable interactions were selected, for synthesis by MedPharma Partners. All compound synthesized are shown in Table 2.

### 2.3. Cloning and Purification of Recombinant Vezf1

**Figure 4:**
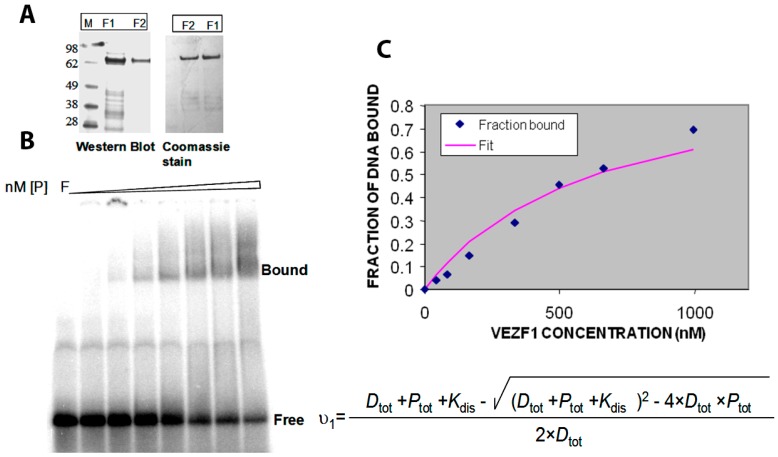
(**A**) His tagged Vezf1 was purified with affinity chromatography using Ni-NTA column. F1 and F2 represent the eluted fractions 1 and 2 which are on SDS PAGE stained with Commassie blue. The integrity of the recombinant protein was tested by Western blot probed with anti-His antibody. (**B**) EMSA (electrophoretic mobility shift assay) was performed to determine the DNA binding constant of Vezf1. 25 nM of radiolabeled DNA were incubated with increasing amount of purified Vezf1 protein 50–1000 nM). (**C**) The band intensities of the free and bound fractions were measured using Image Quant L software in Typhoon Imager. The data were fitted into an equation of binding equilibrium to determine binding constant of 640 nM.

The Vezf1 gene was cloned to encode an N-terminal His6-tag fusion protein into pQE10 (Qiagen, Hilden, Germany). XLBlue (MRF, T7) *E. coli* cells, transformed with pQE10 Vezf1, were grown at 32 °C in 500 mL of LB medium containing 75 mg/mL ampicillin. Protein expression was induced at a cell density of 0.3 A600 nm by the addition of 1 mM IPTG and the cells were grown for an additional two hours at 30 °C. All purification steps were carried out at 4 °C. Since the Vezf1 protein turned out to be susceptible to proteolysis, the purification was carried out in the presence of Protease Inhibitor Cocktail (Roche, Basel, Switzerland) in the sonication buffer. The Vezf1 concentration was estimated from Coomassie blue-stained SDS-PAGE gels using protein standards of known concentration and Western blots were carried out using an anti-His6-tag antibody (Abcam, Cambridge, UK), according to the instructions of the supplier (see Figure 4A).

### 2.4. Determination of DNA Binding Constant Vezf1 to Its Specific DNA Sequence

In order to perform the biochemical testing of the potential small molecule inhibitors, we first determined the DNA binding constant of the recombinant His-Vezf1. Gel mobility shift analysis was used to analyze the interactions of His-Vezf1 recombinant protein with 32P-labelled oligonucleotides containing Vezf1 binding sites characterized at the chicken beta-globin insulator element. Binding assays were carried by incubating 25 nM of radiolabeled oligonucleotides with two-fold increasing concentration of protein ranging from 50–1000 nM in the binding buffer. Protein bound DNA runs as a slower species on the gel (see Figure 4B). The band intensities are used to determine the ratio of bound to unbound nucleic acid on the gel which reflects the fraction of free and bound probe molecules as the binding reaction enters the gel. The data were fitted to the following expression which directly follows from the definition of a bimolecular binding equilibrium and was used to determine the binding constant (*K*_dis_) of Vezf1:

$$v_{1}=\frac{D_{tot}+P_{tot}+K_{dis}-\sqrt{{\left(D_{tot}+P_{tot}+K_{dis}\right)}^{2}-4\times D_{tot}\times P_{tot}}}{2\times D_{tot}}$$

*v*_1_ is the fractional degree of saturation of the available protein binding sites on the DNA at increasing protein concentrations. The dissociation constant of Vezf1 as shown in Figure 4C was approximately 640 nM.

### 2.5. Effect of Small Molecule Inhibitors on DNA Binding Property of Vezf1

**Figure 5:**
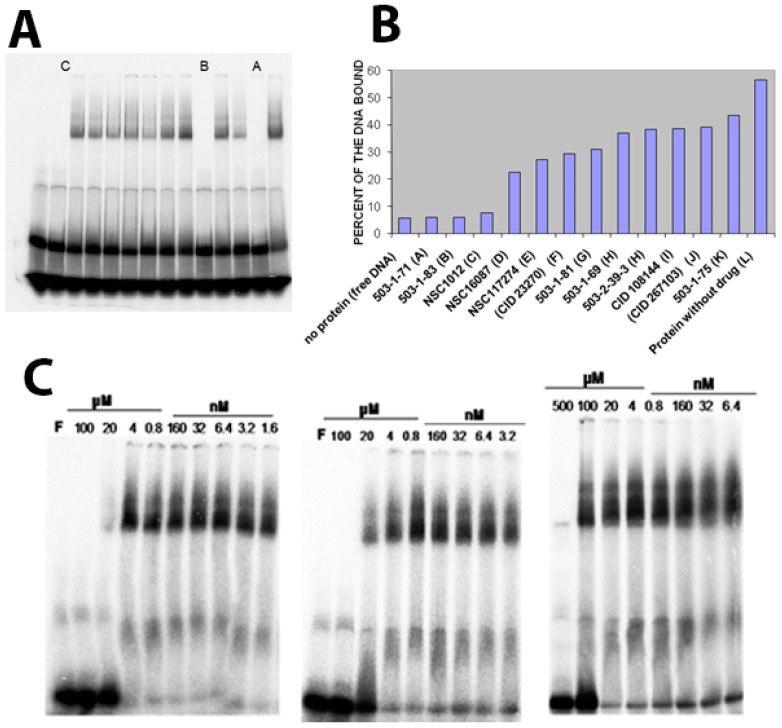
(**A**) EMSA showing the effect of candidate inhibitors on the DNA binding affinity of Vezf1. The protein was pre incubated 500 mM of potential inhibitors for 10 min before adding the DNA to the binding reaction. Compounds noted as A, B and C. Presence of compounds have varying effect on the DNA binding ability of VEZF1. 3 of the 12 tested compounds, A, B and C, severely affected DNA binding of the recombinant Vezf1. (**B**) The bound and free reactions were quantified and plotted as percent bound. A, B and C compounds were identified as 501-1-71, 503-1-83 and NSC1012 respectively. (**C**) To determine the minimum concentration at which these 3 drugs were effective, DNA binding assays were done by pre-incubating 640 nM Vezf1 with varying concentrations of the 3 selected inhibitors, followed by DNA binding assay. As shown, 503-1-83 was most effective in blocking DNA binding by Vezf1 at 20 μM, whereas 503-1-71 showed similar affect at about 100 μM and NSC1012 at 500 μM.

Next, we tested the effect of candidate inhibitors (see Table 1) on the DNA binding affinity of Vezf1. Vezf1 protein at 640 nM concentration was incubated with 500 μM of potential inhibitors for 10 min. All the 12 compounds tested are soluble in water and/or DMSO. 25 nM labeled DNA was added to the reaction and binding assays were done as described above (see Figure 5A). The bound and the free DNA fractions were quantified and plotted (see Figure 5B). Various compounds had varying effect on the DNA binding ability of Vezf1. 3 of the 12 tested compounds, A, B and C, had the greatest degree of potency resulting in a near complete loss of DNA binding by Vezf1. The percent bound DNA is also shown in Table 1.

Next we determined the minimum concentration at which these 3 compounds were effective, and determine the IC50. DNA binding assays were done by pre-incubating 1 μM Vezf1 with varying concentrations of the inhibitors and test the ability of Vezf1 to bind DNA using the gel shift assays. Out of the 3 small molecule inhibitors, T4 (503-1-83) was most potent. It was able to block DNA binding by Vezf1 effectively at a concentration of 20 μM. T6 (503-1-71) and NSC1012 inhibited DNA binding by Vezf1 at concentrations of 100 μM and 500 μM respectively (see Figure 5C).

### 2.6. Effect of Small Molecule Inhibitors on Cell Viability

**Figure 6:**
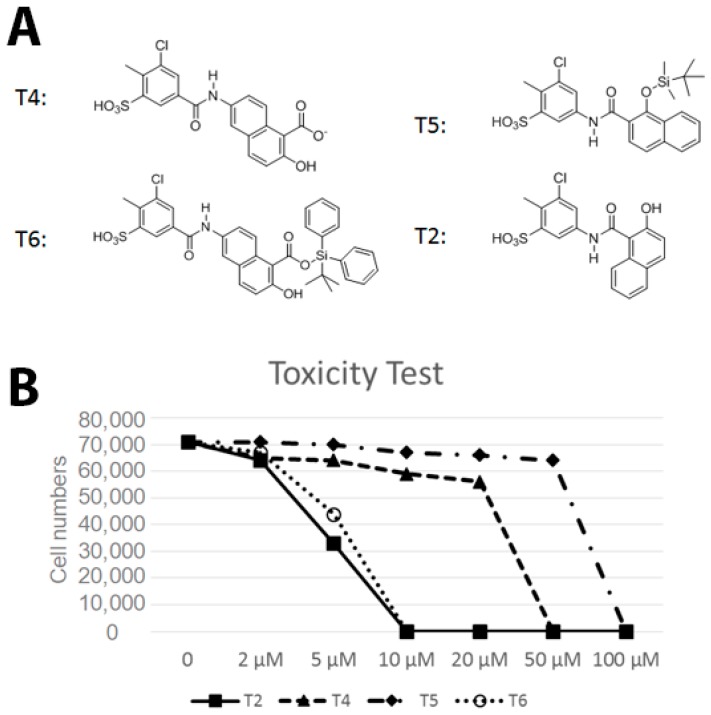
Effect of small molecule inhibitors on cell viability. (**A**) To determine the minimum concentration at which the small molecule inhibitors can be tolerated by cells, four inhibitors were chosen based on their effect on the DNA binding by Vezf1 in gel shift assays. (**B**) MSS31 cells were treated with inhibiots for 24 h and live cells were counted using Trypan blue exclusion test and plotted against the concentration of the inhibitors used. Whereas T5 and T6 were deleterious at very low concentrations, T4, which as a lowest IC50 of 20 µM, was tolerated by cells in this range.

Following the biochemical tests, we wanted to test the ability of these compounds to inhibit Vezf1 activity in cells. However, we first tested the cellular toxicity of some selected compounds. We tested four candidates: T4: most potent based on IC_50_, followed by T6 and T5. We also tested T2 as a control which has no effect in vitro on Vezf1 DNA binding but it has a similar structure to T4 (see Figure 6A). Due its critical role in endothelial development and angiogenesis, we used the mouse endothelial cell line, MSS31, for these studies. Cultured MSS31 cells were treated with increasing concentration of the four inhibitors, T4, T5, T6 and T2 for 24 h. Cells were collected and the live cell population was counted by Bio-Rad cell counter using Trypan Blue exclusion test. Live cells are impermeable to Trypan blue dye, which only stains the dead population. The Bio-Rad cell counter identifies and counts only the unstained cells. As shown in Figure 6B, exposure to T2 and T6 causes cell death at very low concentration. T4 is less toxic and can be tolerated until 50 μM. These data show that T4 can be tolerated by cells at concentrations close to its estimated IC50.

### 2.7. T4 Treated MSS31 Cells Are Incapable of Tube Formation in Matrigel™

**Figure 7:**
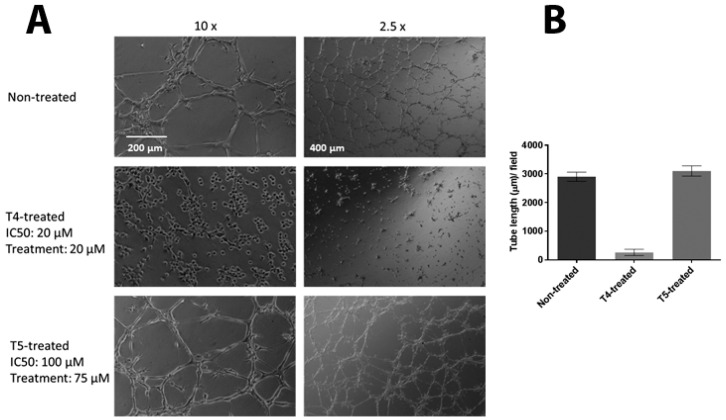
Effect of Vezf1 inhibitors on its biological activity. (**A**) MSS31 cells were treated with 20 µM compound T4 or 75 µM compound T5 for 24 h. Cells were placed on Matrigel™, and tube formation was evaluated after 18 h by bright field microscopy. T4 treatment clearly prevents tube formation in MSS31 cells compare to non-treated and T5-treated cells. 10× and 2.5× show two magnifications of cells in Matrigel™. (**B**) Tube length in A was measured using Image Quant L software and plotted. T4 treated cells show no tube formation whereas T5 treated cells, the tube length is similar to that of the untreated cells.

Vezf1 has a critical function in differentiation of endothelial cells and angiogenesis. Angiogenesis can be modeled in tissue culture by plating MSS31 endothelial cells in VEGF-A containing Matrigel™. The cells arrange into distinct tube like structures within 6–10 h. To determine the ability of the small molecule inhibitors to disrupt this biological activity of Vezf1, we tested the effect of T4 and T5 on the tube formation by endothelial cells MSS31. We exposed MSS31 cells to T4 at 20 μM, a concentration close to its estimated IC_50_ value and T5 at 75 μM, given at 100 μM it is lethal for 24 h. Treated and untreated MSS31 cells were plated in Matrigel™. Within 6–10 h the untreated cells organized into networks as shown by phase contrast microscopy (see Figure 7A). The cells treated with T5 were not at all affected and formed networks similar to untreated cells, however the network formation by cells treated with T4 were strongly inhibited. The tube length was measured which shows a clear absence of tube formation by T4 treated MSS31 cells (see Figure 7B).

These data strongly support T4 to be inhibitor of Vezf1 activity in endothelial cells and has ability to block angiogenesis at non-toxic concentrations.

## 3. Discussion

Multiple signaling pathways that regulate proliferation and differentiation of cells converge into a small subset of transcription factors (TFs) which in turn drive oncogenic transformation by controlling the expression of a large number of genes. This makes TFs compelling targets for cancer therapy. The well-known cancer target TF include MYC, the ETS family, STAT, Fos, Jun, Myb, Sox2 and more. Indirect targeting of TF’s has been used as a therapeutic strategy though inhibition of upstream signaling pathways including kinases. Direct inhibition by small molecule inhibitors against TFs works by blocking their site of activation or the interaction or TF with DNA. These small molecules could be purified from natural source or by high throughput screening of thousands of synthetic compounds using biological assays facilitated by the repository of more than 400,000 candidate drugs at National Cancer Institute. Designing molecules that interrupt DNA binding by TFs has drawbacks since DNA is negatively charged and charged molecules have low permeability. Frequently, small molecules have low affinity and low specificity therefore; they must be used at high doses to achieve therapeutic effects [22]. An example of direct inhibition of TFs include ligands such as Tamoxifen, which binds to nuclear receptor protein, the oestrogen receptor [23]. Besides nuclear receptors, there are only few examples of the direct inhibition of TFs by small molecules. These include the inhibition of FoxM1 DNA binding by naturally occurring antibiotic thiostrepton [24]. Small molecules could be designed to target protein-protein interactions and an example is nutlin, which disrupts the interaction between the transcription factor p53 and its negative regulator MDM2 [25].

Angiogenesis is regulated by the activity of several proteins including Vezf1. Several transcription factors, like VEGF-A, VEGF-AR, and ET-1 have been studied and the mechanistic details of their activity in angiogenesis have been worked out [16]. The discovery of Vezf1 as an endothelial transcription factor and its role in angiogenesis is relatively new. Genetic knockout studies revealed the role of Vezf1 in vascularization, however the mechanism of its regulation is not fully understood [6]. Our previous studies have characterized the activity of Vezf1 at chicken b-globin insulator. Insulators are chromatin elements that block the non-specific activity of enhancers by disallowing their interaction with unassigned promoters [26]. However, the mechanism by which Vezf1 regulates gene expression and angiogenesis during development and disease is unknown. Through construction of a new computational model for Vezf1, work here has identified a potential small molecule binding site (occupied by DNA in the X-ray structure used to construct the model) [21] for use in creating novel small molecule drugs capable of inhibiting Vezf1 from binding to its cognate DNA binding site. The structure of Zif268 was used to model the structure of Vezf1 given the high sequence similarity between the zinc fingers of Vezf1 and Zif268. The Zif268 structure was determined in complex with DNA and demonstrates detailed molecular interactions between zinc fingers and DNA backbone. This motif provides a framework to design molecules that can interrupt DNA binding by zinc fingers. Using AlleGrow and docking tools of FLO to design new compounds, and use of and use of NCI Diversity Compound Library, a novel compound was discovered which inhibits binding of DNA at 20 μM concentration. Of particular note, out of 12 designed compounds, identification of such a small molecule inhibitor may be considered a significant achievement.

Discovery of a small molecular inhibitor against Vezf1 will not only have therapeutic use, but can also be employed to understand its mechanism in development and disease. Tumor cells treated with these inhibitors can be tested for proliferation, migration and tube formation. Further this inhibitor can be used to study wound healing in animal models. This new lead compound is also a candidate for the determination of an X-ray crystal structure of the zinc finger/ligand complex. It is also ideal as a starting point for new rounds of design, synthesis and biological evaluation to find medicinal agents interfering with zinc finger/DNA interactions, a new mechanism of action for development of agents against cancer.

## 4. Materials and Methods

### 4.1. Protein Purification and DNA Binding Assays

XLBlue (MRF, T7) *E. coli* cells, transformed with pQE10 Vezf1 were induced using IPTG and two hours later harvested by centrifugation at 6 K RPM. The cells were washed with STE buffer (10 m Tris-HCl (pH 8.0), 0.1 mM EDTA, 0.1 M NaCl) and centrifuged at 6K RPM. The cell pellet was suspended in buffer A (20 mM KPi (pH 7.5), 1 mM EDTA, 0.1 mM DTT, 0.5 M NaCl, 10% (*v*/*v*) glycerol, 20 mM imidazole) and the cells were disrupted by sonication. The insoluble cell debris was removed by centrifugation (60 min, 13,000× *g*). The supernatant was applied onto a Ni-NTA (Qiagen, Mettmann Germany) column (1 mL gel bed) equilibrated with buffer A. After washing with 150 mL of buffer A, the His-Vezf1 was eluted with 5 mL of elution buffer (20 mM KPi (pH 7.5), 1 mM EDTA, 0.1 mM DTT, 0.5 M NaCl, 10% (*v*/*v*) glycerol, 200 mM imidazole). The eluate was dialyzed overnight against storage buffer (20 mM Hepes (pH 7.5), 40 mM NaCl, 1 mM EDTA, 0.2 mM DTT, 20% glycerol) aliquoted and stored at −80 °C. For DNA binding assays, 50 pmoles of oligonucleotide were end-labeled by polynucleotide kinase in presence of γ [32P] ATP. Purified recombinant Vezf1 was allowed to bind to the oligonucleotide containing the Vezf1 binding site in HS4-FPIII. The reaction was carried out in 15 μL volume in binding buffer (20 mM Hepes 7.5, 10 mM MgCl_2_, 50 mM NaCl, 1 mM EDTA, 5 ng/uL poly dA.dT) and incubated for 10 min at room temperature followed by the addition of 5% Ficoll.

The bound and the free DNA was separated on 5% acrylamide gel run in .5XTBE buffer for 2 h at 150 V. The gel was dried and imaged by Typhoon (GE Healthcare Lifesciences, Chicago, IL, USA). The band densities were quantified by the ImageQuant TL software (GE Healthcare Lifesciences). The top strand sequence for the duplex used for gel mobility shift analyses is 5′AGGCGCGCCCCGGTCCGGCGCTCCCCCCGCATCCCCGAGCCGGGGCGCGCCT3′. The relative band intensities were used to fit the data to an equation representing bimolecular binding equilibrium using Microsoft Excel and the binding constant was determined.

### 4.2. Small Molecule Inhibitors

The small molecule synthesized by MedPharma Partners (Boston, MA, USA) were suspended in DMSO at 10 mg/mL concentration. Further dilutions for use in the gel shift assays and cell culture were done in water. The diluted samples were stored in −20 °C whereas the stock in DMSO was stored in 4 °C.

### 4.3. Cell Viability and Tube Formation Assays

MSS31 mouse endothelial cells were collected by trypsinization and counted using a Bio-Rad Cell Counter (Hercules, CA, USA). The cell viability assay was performed plating 1 × 10^6^ MSS31 cells in each well of a 6-well plate, After 24 h the cells were treated with various small molecules by directly adding into the medium at various concentrations. The cells were incubated for 24 h and vaibilty of cells was measured. The cells were trypsanized and treated with Trypan Blue. The live cell population which is stained is counted using Bio-Rad cell counter. The tube formation or sprouting assay was performed by plating 2 × 10^5^ cells on a 24-well plate coated with VEGF-A supplemented Matrigel™ (BD Biosciences, East Rutherford, NJ, USA) according to the manufacturers protocol. The cells were incubated at 37 degrees for 3–18 h. Sprouting was scored using images from phase contrast microscopy. The length of the tubes was measured by Image J software (National Institutes of Health, Bethesda, MA, USA). For inhibitor treatment, MSS31 cells were treated for 24 h with compounds, after which cells were trypisinized and plated on Matrigel™ for tube formation assays.

## Figures and Tables

**Table 1:** Rank order of known compounds using each method. Results are shown of testing docking protocols.

	SDock+	McDock+	SDock+ Plus McDock+
M1012	2	7	3
M16087	3	3	2
M609974	12	15	17

**Table 2:** Summary of the compounds used and their activity.

Compound Structure	Compound Number	% DNA Bound in EMSA (500 μM)	Activity Concentration
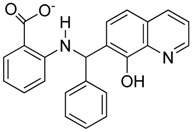	NSC1012 (1)	7.38%	500 μM
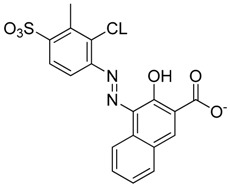	NSC16087 (2)	22.56%	500 μM
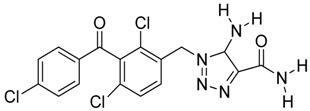	NSC609974 (3)	38.45%	500 μM
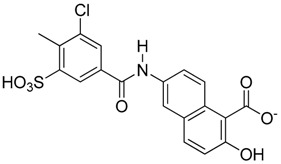	T4	5.94%	20 μM
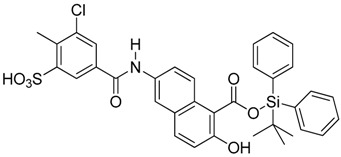	T6	5.92%	100 μM
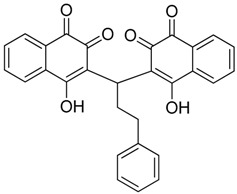	CID 272651 (NSC117274)	27.13%	NA
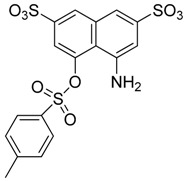	CID 23270	29.37%	NA
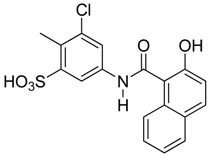	T3	30.81%	NA
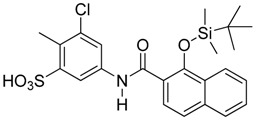	T5	36.81%	NA
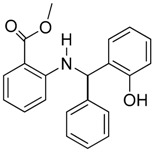	T2	38.23%	
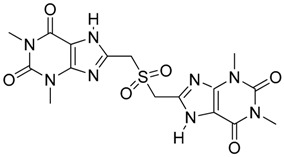	CID 267103	38.95%	
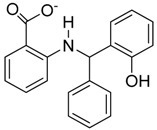	T1	43.58%	

## Supplementary Materials

The following are available online: Figure S1.
