# Supplementary material for: Characterization of Small Molecules Inhibiting the Pro-Angiogenic Activity of the Zinc Finger Transcription Factor Vezf1
Source: Molecules. 2018 Jul 3;23(7):1615. doi: 10.3390/molecules23071615 (PMC6100598; doi:10.3390/molecules23071615)
Supplement: Supplementary file 1 [file molecules-23-01615-s001.pdf]

Characterization of Small Molecules Inhibiting Pro-angiogenic Activity of the Zinc Finger Transcription Factor Vezf1

He Ming[1], Qianyi Yang[4], Allison Norvil[1], David Sherris[3] and Humaira Gowher[1, 2]*

[1]Department of Biochemistry, [2]Purdue University Center for Cancer Research,

Purdue University, West Lafayette, Indiana 47907

[4]Present address: Department of Anesthesiology, Washington University School of Medicine, 660 S Euclid Ave, St. Louis, MO 63110

[3]Present address: GenAdam Therapeutics, Inc., 37 Neillian Crescent, Jamaica Plain, MA 02130

*Corresponding author: hgowher@purdue.edu; 3018202794

Running title: Small molecule inhibition of Vezf1 activity in endothelial cells.

Keywords: Vezf1; angiogenesis; vascular biology; endothelial cells; MSS31, tube formation, small molecule inhibitors computational modeling.

A

B

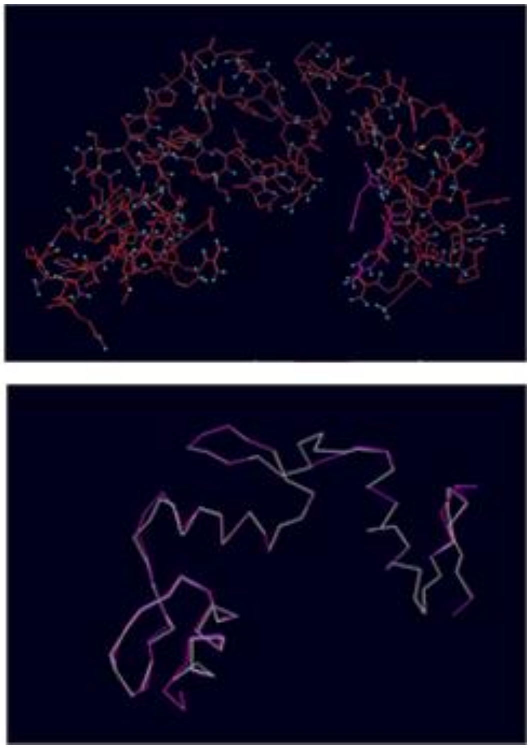

Figure S1: A. Red atoms are held fixed during docking of compounds; purple atoms are allowed restrained movement during the energy minimization step. In B., Model (white) is superimposed onto the crystal structure AAY (purple). Only the alpha carbon trace is shown.
